# Diphtheria and Scarlatina

**Published:** 1883-11

**Authors:** 


### Diphtheria and Scarlatina.

The identity or not of the poisons producing diphtheria and scarlatina has been the subject of much discussion, and any information bearing upon the question is worthy of record. A curious instance of the manner in which these diseases at times coexist and alternate with each other is recorded in a report addressed by Mr. W. H. Power to the Local Government Board, and to which we refer elsewhere, on a prevalence of infectious diseases at Whitstable. Diphtheria commenced in Whitstable in October, 1880, and continued till January in the following year. It had not long prevailed when scarlatina appeared, the two diseases being concurrent and attacking at one time different members of the same family. The diphtheria then began to disappear, whilst the scarlatina became more prevalent and assumed an increasingly fatal type. Towards the middle of 1881 the scarlatina epidemic declined and diphtheria, at times fatal, reappeared; indeed, with the absolute disappearance of scarlatina, diphtheria, early in 1882, steadily spread, remaining more or less prevalent throughout the year. During these several occurrences more than one of the medical practitioners in attendance on the cases had difficulty in diagnosing between the two diseases; thus cases of smart throat illness associated with distinct skin rash and altogether free from faucial false membrane occurred, and yet at no period of the illness or convalescence did any such tendency to desquamation, as usually follows on scarlatina, show itself. Eight or ten years ago very similar circumstances were observed at Whitstable, diphtheria being exceptionally fatal and at the same time associated with a prevalence of scarlatina. Mr. Power abstains from expressing any comment on the questions arising from a consideration of these circumstances; the facts are, however, highly interesting.—*The Lancet.*

